# Oligoclonal Bands in Cerebrospinal Fluid of Black Patients with Multiple Sclerosis

**DOI:** 10.1155/2015/217961

**Published:** 2015-07-29

**Authors:** Paulo Diniz da Gama, Luís dos Ramos Machado, José Antonio Livramento, Hélio Rodrigues Gomes, Tarso Adoni, Rogério de Rizo Morales, Rodrigo Assad Diniz da Gama, Daniel Assad Diniz da Gama, Marco Aurélio Lana-Peixoto, Yara Dadalti Fragoso, Dagoberto Callegaro

**Affiliations:** ^1^Pontifical Catholic University of São Paulo, Rua Conde Fco Matarazzo 58, 18030-010 Sorocaba, SP, Brazil; ^2^University of São Paulo, São Paulo, SP, Brazil; ^3^Hospital Sírio-Libanês, São Paulo, SP, Brazil; ^4^Federal University of Uberlândia, MG, Brazil; ^5^Federal University of Minas Gerais, Belo Horizonte, MG, Brazil; ^6^Metropolitan University of Santos, Santos, SP, Brazil; ^7^Demyelinating Diseases Reference Center, Hospital das Clinicas, University of São Paulo, São Paulo, SP, Brazil

## Abstract

Genetic susceptibility is a well-recognized factor in the onset of multiple sclerosis (MS). The objective of this study was to determine the frequency of oligoclonal bands (OCB) restricted to the cerebrospinal fluid, in an ethnically mixed group of MS patients in the city of São Paulo, Brazil. Techniques used to detect OCB consisted of isoelectric focusing followed by immunoblotting. OCB were found in 49 (54.4%) out of 90 patients with clinically definite MS; out of the 23 brown/black patients, 17 (73.9%) were OCB+; out of the 66 white patients, 32 (48.5%) were OCB+; and the only patient yellow was OCB+ (*p* = 0.05). Analysis of the IgG index was also consistent with the findings, but with lower statistical significance. The data presented in our study show that the ethnic differences in MS extend to the immune response.

## 1. Introduction

Multiple sclerosis (MS) is a chronic inflammatory demyelinating disease of the central nervous system. MS is thought to develop in genetically susceptible individuals when one or more environmental factors trigger a cascade of events leading to disease manifestation [1]. Genetic susceptibility is a well-recognized factor in the onset of MS [1]. Individuals of African origin appear to have a “genetic protection” against MS, as reflected by the low incidence of the disease among Africans and Afrodescendants [1, 2]. This holds true not only for people living in Africa, but also for those living in Europe, North America, and South America who have an African background [3–5]. Since this ethnic factor seems to influence the physiopathogenic mechanisms of MS, it is plausible to expect that inflammatory markers of the disease might also be influenced by ethnicity. Here, we describe the frequency of oligoclonal bands (OCB) and IgG index status in cerebrospinal fluid (CSF) in an ethnically mixed group of MS patients in the city of São Paulo, Brazil.

## 2. Methods

The present study was approved by the Research Ethics Committee of the University of São Paulo, under the number 800/05. All patients or their legal guardians signed the informed consent agreement prior to enrolment in this study. The MS patients' histories were taken, physical examinations were conducted, and MRI analysis was done immediately after the patients had signed their consent. A second medical doctor reevaluated these parameters independently. Both doctors were neurologists with expertise in MS diagnosis (authors PDG and DC). Subsequently, the patients underwent sampling of CSF as part of the laboratory investigation of MS.

The diagnosis of MS was established in accordance with McDonald's International Criteria, as revised in 2005 [6]. None of the patients had previously undergone a CSF exam and, therefore, the results from the CSF analysis were not used to establish the diagnosis of MS, excluding thereby a diagnostic interpretation bias. Patients who failed to fulfill the clinical and radiological criteria for diagnosing MS were excluded from the present study. In accordance with ethical considerations, they were guaranteed full follow-up at the institution's department of neurology, irrespective of whether they were participating in this study.

A group of 39 patients with a variety of chronic inflammatory disorders of the CNS and 19 subjects with neither neurological complaints nor infectious conditions, whose CSF sample was collected during anesthetic procedure for minor surgery, were used as controls.

The criteria used for ethnic origin were the same as used by the Brazilian research institute, the IBGE (Brazilian Institute of Geography and Statistics). Because of the high degree of miscegenation in Brazil, the IBGE classifies races through self-declared skin color, grouped as white, black, brown (mulatto/mixed), “yellow” (East Asian), and indigenous Indian.

Patients with MS and both control groups were recruited consecutively, all during the same period, from August 2005 to January 2008.

Blood serum and CSF analysis were carried out simultaneously. The CSF analysis included the classical routine tests (cytomorphological profile, determination of total protein content, and assaying of glucose and chlorides). Upon suspicion of involvement of infectious processes, specific immunological reactions were performed and, possibly, analysis on antigenic material and PCR.

Isoelectric focusing (IF) on polyacrylamide gel, followed by immunoblotting [7], was used to assess OCB (ETC Elektrophorese Technik, Westermeier & Schickle GmbH, Bahnhofstrasse 26, 72138 Kirchentellinsfurt, Germany). Each sample of CSF and blood serum was subjected to IF (CWP-400 Isolab Inc.), always with parallel samples. In each of the procedures, a positive and a negative control sample were used. After IF, the proteins were transferred to a nitrocellulose membrane (Bio Agency) for immunoblotting method entailing a primary antibody (goat anti-human IgG, Sigma) and a peroxidase-labeled secondary antibody (polyclonal rabbit anti-goat immunoglobulins, Dako Cytomation). OCB were considered positive when two or more bands were found in the CSF, but absent in the serum. For greater reliability, the results from these tests were examined by two specialists (authors JAL and HRG) who were not aware of the group to which the samples belonged (MS or controls). If there was any doubt or difference of opinion regarding the interpretation of a particular result, the sample in question was processed again.

Quantitative intrathecal immunoproduction of IgG was carried out in parallel. Concentrations of IgG and albumin both in the serum and CSF were measured by means of nephelometry. This made it possible to determine the IgG index, which was considered to be greater than normal for values ≥0.8.

Tests aimed at ruling out diseases that might have differential diagnoses with MS in specific cases were performed when deemed necessary [8].

## 3. Results

Preliminary results were presented earlier [9, 10].

The cohort comprised 90 subjects with MS who were attending the Demyelinating Diseases Reference Center at Hospital das Clinicas, University of São Paulo Medical School.

**Figure 1:**
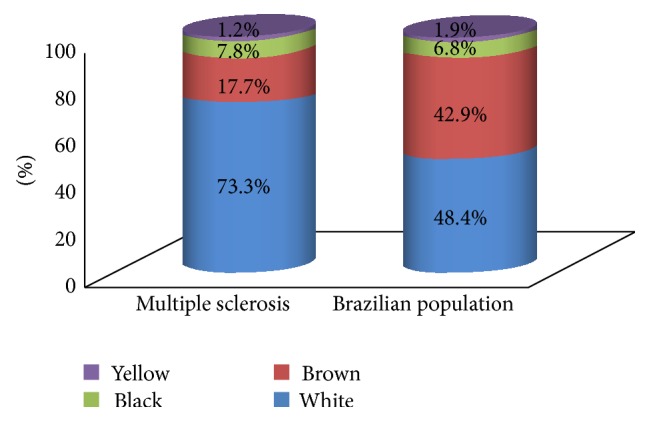
Comparison of self-declared skin color between the study population with multiple sclerosis and the general Brazilian population.

Figure 1 shows the demographic characteristics of the population studied. From this group, of 90 patients with MS, 66 patients self-declared themselves as white, 23 as brown or black, and one as yellow. The designations of black and brown (mulatto/mixed) both refer to individuals of African origin and therefore were grouped as a single element in subsequent analysis.

Out of the 90 patients with MS, 49 (54.4%) presented OCB restricted to the CSF. The specificity of OCB in the CSF was 100% when patients without neurological disease were used as controls and was 82.1% when patients with inflammatory diseases of the CNS were used as the control.

**Figure 2:**
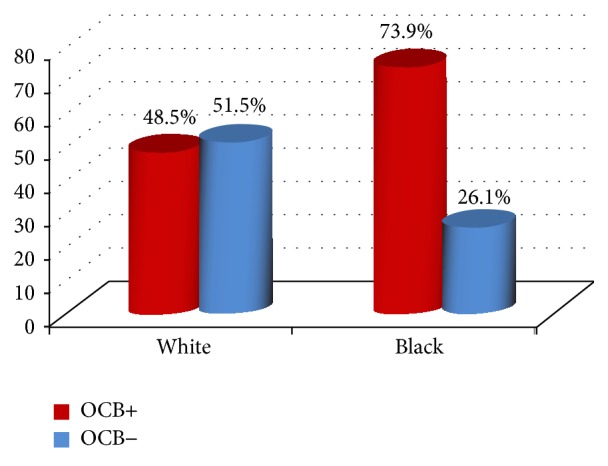
OCB (oligoclonal bands). The test of nonassociation between color and presence of OCB gave the following results: *X* ^2^ = 3.54; g.l. = 1; *p* = 0.051.

When we compared the self-declared ethnicity (skin color) with the results from the OCB analysis, it was observed that (1) out of the 23 brown/black patients 17 (73.9%) were OCB+; (2) out of the 66 white patients 32 (48.5%) were OCB+; and (3) the only patient self-declared as yellow was OCB+. There was a positive correlation between African origin and OCB presence in the CSF (*X* ^2^ = 3.54; g.l. = 1; *p* = 0.051; *R* = 0.69; 95% CI = 0.48–0.98; Figure 2).

The IgG index was abnormal in 47 of the 90 patients with MS (52.2%), with a median of 0.8 and mean of 1.02 (SD 0.67). The sensitivity of the IgG index for diagnosing MS was 52.2%; the specificity was 94.8% when patients without neurological disease were used as controls and was 64.2% when patients with inflammatory diseases of the CNS were used as controls.

**Figure 3:**
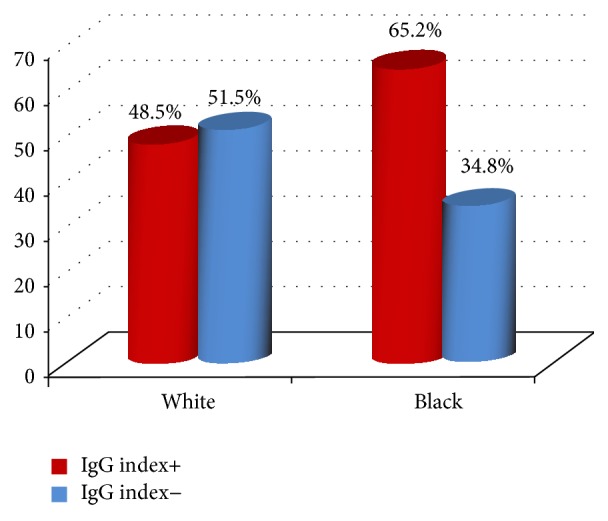
IgG index+ (≥0.8). The test of nonassociation between color and IgG+ gave the following results: *X* ^2^ = 1.39; g.l. = 1; *p* = 0.24.

When we compared the self-declared ethnicity with the results from the IgG index analysis, it was observed that (1) out of the 23 brown/black patients 15 (65.2%) had an abnormally high IgG index; (2) out of the 66 white patients 32 (48.5%) had an abnormally high IgG index; and (3) the only patient who was self-designated as yellow had a normal IgG index (*X* ^2^ = 1.39; g.l. = 1; *p* = 0.24; Figure 3).

**Figure 4:**
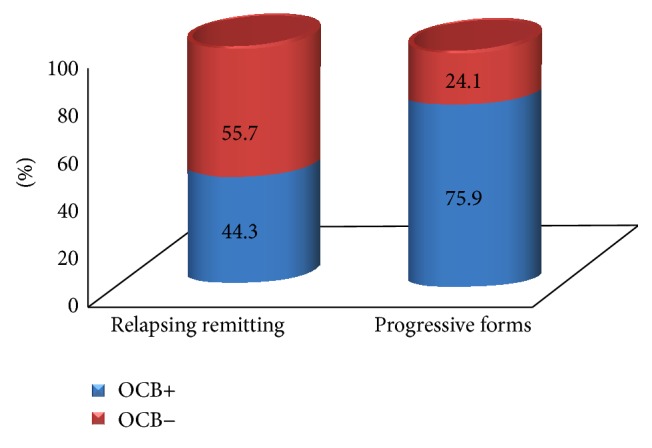
Distribution of the presence of oligoclonal bands (OCB) according to the clinical forms of multiple sclerosis. Test of homogeneity: *X* ^2^ = 7.913; g.l. = 1; *p* = 0.0049; relative risk *R* = 0.58; 95% CI = 0.41–0.83.

**Figure 5:**
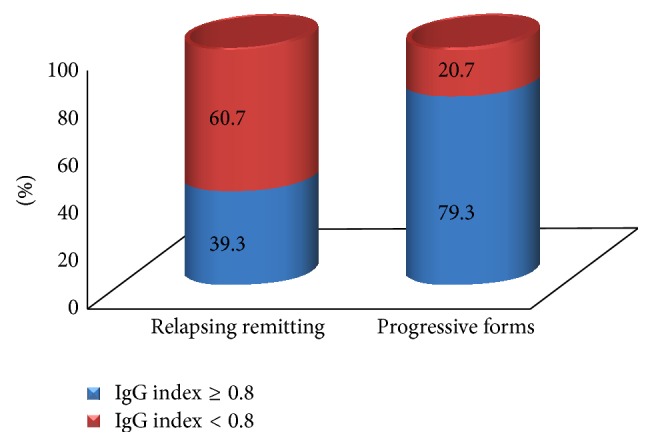
Distribution of the IgG index status according to the clinical forms of multiple sclerosis. Test of homogeneity: *X* ^2^ = 12.58; g.l. = 1; *p* = 0.0004; relative risk *R* = 0.50; 95% CI = 0.35–0.71.

**Figure 6:**
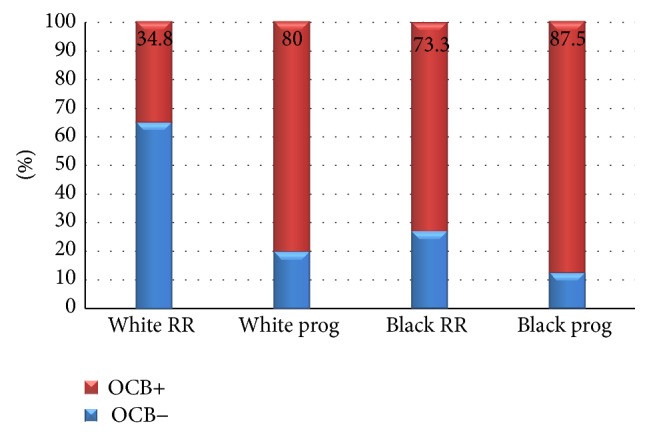
RR (relapsing remitting); prog (secondary-progressive, primary-progressive, and progressive relapsing forms of multiple sclerosis); OCB (oligoclonal bands). Association between OCB and clinical forms of multiple sclerosis adjusted according to patients' self-declared color. Test of nonassociation: *X* ^2^ = 7.40; g.l. = 1; *p* = 0.0065.

When OCB were analyzed in relation to MS disease clinical forms, it was observed that progressive forms of MS were significantly related to OCB and IgG index status in CSF (Figures 4 and 5). This result was remarkable when adjusted for ethnicity. From the group of patients with the relapsing-remitting form of MS, 16 white patients (out of 46; 34.8%) were OCB+, while 17 brown/black patients (out of 23; 73.3%) were OCB+. When progressive forms of MS were taken into consideration, 16 white patients (out of 20; 80%) were OCB+, while seven black patients (out of eight; 87.5%) were OCB+. This result showed that there was a positive correlation between the presence of OCB and the progressive course of MS when adjusted for ethnicity (*X* ^2^ = 7.40; g.l. = 1; *p* = 0.006; Figure 6).

**Figure 7:**
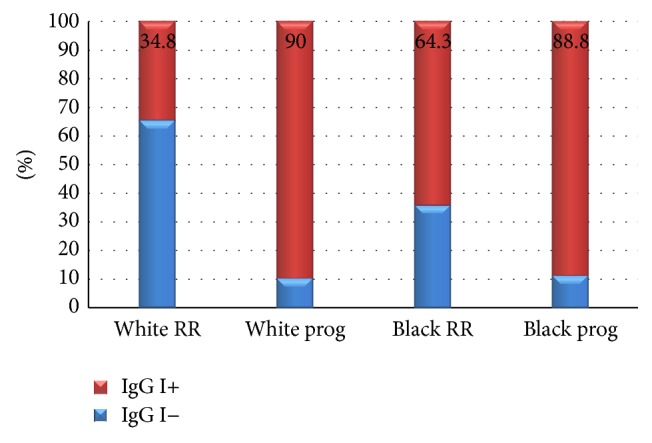
RR (relapsing remitting); prog (secondary-progressive, primary-progressive, and progressive relapsing forms of multiple sclerosis); IgG I (IgG index). Association between IgG index status and clinical forms of multiple sclerosis adjusted according to patients' self-declared color. Test of nonassociation: *X* ^2^ = 7.7; g.l. = 1; *p* = 0.0055.

From the group of patients with the relapsing-remitting form of MS, 16 white patients (out of 46; 34.8%) had an abnormally high IgG index, while 16 brown/black patients (out of 23; 69.6%) had an abnormally high IgG index. When progressive forms of MS were taken into consideration, 18 white patients (out of 20; 90%) had an abnormally high IgG index, while eight black patients (out of nine; 88.8%) had an abnormally high IgG index. This result showed that there was also a positive correlation between the presence of an abnormally high IgG index and a progressive course of MS when adjusted for ethnicity (*X* ^2^ = 7.70; g.l. = 1; *p* = 0.0055; Figure 7).

Other correlations, such as disease duration and disease progression and/or relapse rate, were not assessed due to the very wide range of these parameters in this group of 90 patients.

## 4. Discussion

MS is widely considered to be an autoimmune disease due primarily to CD4+ T-cell mediated immune responses to the major myelin proteins, myelin basic proteins (MBP) and proteolipid proteins (PLP) [1]. Humoral immune responses are also believed to contribute to the immunopathology of MS, and the presence of OCB and/or increased IgG index in CSF directly reflects a high humoral inflammatory response in the patient [11]. In the present study, the presence of OCB in CSF was significantly associated with African origin among the patients and with progressive forms of MS in all ethnicities. The presence of OCB in CSF has been correlated with more aggressive forms of MS [11] and with higher risks of conversion to MS from clinically isolated demyelinating syndrome [12, 13]. Furthermore, African ethnicity has been correlated with a more severe course of MS [3, 4, 14–16] and higher humoral activity [17].

Large studies that investigated genetic differences and clinical and demographic characteristics in relation to OCB status and IgG index have strongly supported the idea that MS patients with and without OCB and/or abnormally high IgG index are genetically distinct [18, 19]. This differentiation may extend to races, as demonstrated through our data.

The aim of the present study was not to assess the correlation between OCB and disease severity or progression in patients of different ethnicities but rather to investigate whether there would be any difference in the immune response in CSF depending on ethnicity. However, the significantly higher humoral activity in MS among patients with an African background indicates possible (and still unknown) biological differences in MS depending on the ethnic background.

The data presented in our study show that the ethnic differences in MS extend to the immune response and may add another measurable immunological phenomenon to the list of differences between ethnic groups.
